# Orbital shaking conditions augment human nasoseptal cartilage formation in 3D culture

**DOI:** 10.3389/fbioe.2024.1360089

**Published:** 2024-03-15

**Authors:** Thomas Harry Jovic, Feihu Zhao, Henry Jia, Shareen Heather Doak, Iain Stuart Whitaker

**Affiliations:** ^1^ Reconstructive Surgery & Regenerative Medicine Research Centre, Swansea University, Swansea, United Kingdom; ^2^ Welsh Centre for Burns & Plastic Surgery, Morriston Hospital, Swansea, United Kingdom; ^3^ Department of Biomedical Engineering & Zienkiewicz Institute, Faculty of Science and Engineering, Swansea University, Swansea, United Kingdom; ^4^ Swansea University Medical School, Swansea University, Swansea, United Kingdom

**Keywords:** cartilage, tissue engineering, chondrogenesis, dynamic culture, computational modelling

## Abstract

**Introduction:** This study aimed to determine whether a dynamic orbital shaking culture system could enhance the cartilage production and viability of bioengineered nasoseptal cartilage.

**Methods:** Human nasal chondrocytes were seeded onto nanocellulose-alginate biomaterials and cultured in static or dynamic conditions for 14 days. Quantitative polymerase chain reaction for chondrogenic gene expression (type 2 collagen, aggrecan and *SOX9*) was performed, demonstrating a transient rise in *SOX9* expression at 1 and 7 days of culture, followed by a rise at 7 and 14 days in Aggrecan (184.5-fold increase, *p* < 0.0001) and Type 2 Collagen (226.3-fold increase, *p* = 0.049) expression. Samples were analysed histologically for glycosaminoglycan content using Alcian blue staining and demonstrated increased matrix formation in dynamic culture.

**Results:** Superior cell viability was identified in the dynamic conditions through live-dead and alamarBlue assays. Computational analysis was used to determine the shear stress experienced by cells in the biomaterial in the dynamic conditions and found that the mechanical stimulation exerted was minimal (fluid shear stress <0.02 mPa, fluid pressure <48 Pa).

**Conclusion:** We conclude that the use of an orbital shaking system exerts biologically relevant effects on bioengineered nasoseptal cartilage independently of the expected thresholds of mechanical stimulation, with implications for optimising future cartilage tissue engineering efforts.

## 1 Introduction

There is a clinical need for cartilage tissue engineering owing to the poor inherent regenerative capacity of human cartilage (Jessop et al., 2017). Disease processes such as malignancy, trauma and congenital defects may render facial structures devoid of suitable cartilage to maintain form and function and usually require the use of autologous grafting in order to enable reconstruction (Jessop et al., 2017). As such, there has been increasing interest in the ability to generate facial cartilage *in vitro* to address the need for donor tissue in addressing the burden of these disease processes.

Whilst there has been significant interest in scaffold development and cell sources for cartilage tissue engineering, of equal importance is the optimisation of the culture conditions (Almouemen et al., 2019). The environment in which tissue engineered cartilage is cultivated incorporates culture conditions such as media constituents, growth factors and exposure to various stimuli including mechanical and electrical stimulation (Al-Himdani et al., 2017). These cues can dictate the efficiency of cell growth needed to populate a scaffold and moreover direct chondrocytes towards the production of cartilage extracellular matrix, both of which are essential for achieving the necessary biomimicry for clinical implementation.

Chondrocytes are recognised to be a mechanosensitive cell type, particularly in articular cartilage, and extrinsic stimuli may be converted into a mechanotransduction signal to increase chondrogenesis and/or proliferation which can be exploited *in vitro* (Lane Smith et al., 2000; Millward-Sadler and Salter, 2004; Shahin and Doran, 2015). Mechanical stresses play a role at all stages of cartilage development: from chondrogenesis to maturation and homeostasis, and are detected by deformation of the pericellular matrix and cell surface receptors, such as integrins, channelomes and the primary cilium, leading to the induction of gene expression that directs extracellular matrix production (Zhao et al., 2020). The nature of the stress may evoke cartilaginous or mineralised matrix as a response highlighting the importance of appropriate biomechanical signals to the chondrocyte and pericellular matrix (Gao et al., 2014; Zhao et al., 2020). Forces such as hydrostatic pressure, shear stress, tensile stress and compressive stress can be applied continuously, intermittently or cyclically to emulate a large range of forces that may be endured *in vivo* (Heath, 2000). Studies of articular cartilage have demonstrated that cyclic compressive loading, as seen on load bearing joints, is an important contributor to cartilage homeostasis, but can have deleterious effects when excessive, leading to osteoarthritic changes (Havaldar et al., 2014; Young et al., 2017; Takeda et al., 2021). Similarly, tensile and hydrostatic forces have been demonstrated to be important contributors to cartilage ECM production at physiological levels, which in excess, can drive osteogenic changes and mineralisation of matrix (Elder and Athanasiou, 2009; Bleuel et al., 2015; Zhong et al., 2018; Maki et al., 2021).

The ability to control the environment in regard to mechanical forces has as such promoted the development of dynamic culture conditions in the form of bioreactor systems that attempt to maximise extracellular matrix production in tissue engineered cartilage (Li et al., 2017; Sharifi and Gharravi, 2019; Fu et al., 2021; Paggi et al., 2022). Whilst it is accepted that cartilage cells demonstrate superior phenotypic retention when cultured in 3D compared to 2D (Caron et al., 2012), growth can be further optimised according to a number of extrinsic factors such as perfusion with growth media, exposure to certain types of mechanical stimulation and supplementation with growth factors: many of these concepts underpin the design of bioreactors for enhancing tissue engineering (Concaro et al., 2009; Li et al., 2017). As such, bioreactors are an attractive means of maximising environmental control, particularly in the realm of *de novo* tissue engineering (Martin et al., 2004). Whilst most research has focussed on articular cartilage, there are a limited number of studies that indicate facial cartilages also have the capacity to respond favourably to hydrostatic pressure and compressive loading, but that these responses may differ between different facial cartilages such as temporomandibular and nasoseptal cartilage (Takano-Yamamoto et al., 1991; Candrian et al., 2008; Correro-Shahgaldian et al., 2016).

In particular, culturing cells in dynamic conditions may replicate biomechanical signals such as compression and perfusion, normally encountered in the development of cartilage tissue, and as such facilitate cell growth and matrix production (Concaro et al., 2009; Fu et al., 2021). However, the effect of simple perfusion-based bioreactors for nasoseptal cartilage tissue engineering is less well explored than its articular counterpart (Li et al., 2017).

As such, this study aimed to assess the efficacy of an orbital shaking culture system in enhancing *in vitro* nasoseptal cartilage bioengineering. Through the use of *in silico* modelling of the dynamic culture conditions, we sought to determine the nature of the stimuli exerted upon the chondrocyte-biomaterial constructs to elucidate their contribution towards cartilage production, proliferation and cell viability with the ultimate aim to facilitate *de novo* cartilage biomimicry.

## 2 Materials and methods

### 2.1 Isolation and culture of human nasoseptal cartilage cells

Human nasoseptal chondrocytes (hNSCs) were acquired directly from excess nasoseptal cartilage discarded from patients undergoing septorhinoplasty procedures with informed consent obtained from all patients. All methods were performed in accordance with relevant guidelines and regulations as approved by the Research Ethics Committee, National Institute for Social Care and Health Research, Welsh Government (IRAS ID 99202). Cartilage tissue was diced into 1 mm^3^ pieces in aseptic conditions and digested in 0.4% pronase (Roche, UK) for 1 h at 37°C, 5% CO_2_ with gentle agitation, followed by secondary digestion in 0.2% collagenase type I solution for 16 h (Sigma-Aldrich, Poole, UK). The resultant cell suspension was filtered through a 40 μm cell strainer (Corning, NY, USA) and centrifuged at 350 g for 6 min. Cells were cultured in 5% CO_2_ at 37°C with culture medium changed every 2–3 days. Culture medium (chondromedia) comprised Dulbecco’s Modified Eagle Medium without glucose (Sigma-Aldrich, Poole, UK) supplemented with 10% fetal bovine serum (Sigma-Aldrich, Poole, UK), 100 μg/mL penicillin and 100 U ml^−1^ streptomycin (Sigma-Aldrich, Poole, UK), 1 mM glucose (SigmaAldrich, Poole, UK), and 0.1% non-essential amino acids (Thermo Fisher Scientific, MA, USA). Low glucose concentrations were used to preserve chondrocyte differentiation and ECM production as previously described (Heywood et al., 2014). Cells were seeded at a density of 7000 cells per cm^2^, grown to 70% confluence over 10–14 days and passaged using 0.05% trypsin-EDTA (Thermo Fisher Scientific, Paisley, UK). Cell number was calculated using a trypan blue exclusion assay (Thermofisher Scientific, MA, USA). Cells were used at Passage 2 from 3 separate patients.

### 2.2 Formation of chondrocyte-biomaterial constructs

A composite nanocellulose blend (NCB) and alginate biomaterial was used to encapsulate the cells in 3D culture. AVAPCO (Thomaston, Georgia, USA) provided the BioPlus^®^ nanocellulose particles that were produced from raw wood chip biomass using AVAP^®^ technology, which fractionates the biomass into cellulose, lignin and hemicelluloses using ethanol and sulphur dioxide as previously described (Nelson and Restina, 2014). NCB gel (Nanocellulose Blend, wt. 3%) in water has been extensively characterised in our previous work (Kyle et al., 2018). Sodium alginate (from brown algae, 80,000–120,000 Da, medium viscosity, 2% at 25°C, Sigma-Aldrich, MO, USA) was UV sterilised in powder form and dissolved in sterile, tissue culture grade water to produce a 2.5% (w/v) solution as previously optimised and described (Al-Sabah et al., 2019; Jessop et al., 2019). 75 mL of NCB was made into a composite biomaterial through the addition of 25 mL of the 2.5% (w/v) sodium alginate solution, consistent with our previous characterisation and optimisation of these composite biomaterials (Kyle et al., 2018; Al-Sabah et al., 2019; Jessop et al., 2019). The composite was then homogenised using a stirrer and syringe and autoclaved at 100 kPa, 121°C for 45 min and corrected to pH 7.4 through the dropwise addition of 1M sodium hydroxide solution.

A cell-biomaterial suspension of 3 × 10^6^ cells per ml was produced, in keeping with our previous work on cartilage bioengineering, allowing for use of lower passage cells and minimizing 2D monoculture expansion (Al-Sabah et al., 2019; Jessop et al., 2019). The suspension was used to dispense 100 μL hemispheres of biomaterial into a sterile mould to ensure geometric consistency. The constructs were crosslinked using 0.5 M calcium chloride as previously reported (Al-Sabah et al., 2019) for 5 min and thereafter washed with warm PBS solution.

### 2.3 Dynamic and static cell culture conditions

Constructs were immersed in media within separate wells of a 48 well plate and cultured for up to 14 days at 37°C and 5% CO_2_ with media changes performed every 3 days.

One plate was added to an orbital shaker, calibrated to 37°C and 5% CO_2_ in the cell incubator and set at 500 rpm. The plate was secured to the orbital shaker with autoclave tape (dynamic condition). Another plate was added to a standard shelf of the same incubator but not subjected to any movement or shaking (static condition). Pauses in the dynamic culture conditions were only implemented for the purposes of media changes and specimen harvesting at 4 h (Day 0), 24 h (Day 1), 7- and 14-day timepoints.

### 2.4 Quantitative polymerase chain reaction

Chondrogenicity of each condition was determined at the level of gene expression through Real Time Quantitative Polymerase Chain Reaction (RT-qPCR). For each conditions, cells from three separate patients (biological repeats) were used with technical triplicates acquired for each condition and time point.

In order to quantify gene expression, triplicates of each condition and timepoint were mixed with 0.1% (w/v) EDTA solution (Sigma Aldrich) and TRIzol (Invitrogen, Thermo Fisher Scientific, MA, USA), frozen at −80°C over 24 h and subsequently degraded with a TissueRuptor II probe for 30 s. The lysate was processed using chloroform, Qiagen QIAshredder and RNeasy Mini Extraction kits (Qiagen, Germany) to yield RNA for reverse transcription. The RNA was precipitated using 70% RNase and DNase were not used. The RNA was quantified and assessed for purity using a Nanodrop Spectrophotometer (Thermo Fisher Scientific, MA, USA) (using 260/230 and 260/280 values > 1.6 only) and stored at −80°C. The concentrations of RNA in each sample were diluted with ultrapure nuclease free water across repeats to yield 11 μL samples each containing 225 ng RNA. The RNA was converted to cDNA through reverse transcription, The samples were added to a T100 Thermal Cycler (BioRad, CA, USA) for 5 min at 65°C and 1 min at 4°^C^. The samples were then each combined with a mixture of 1 µL of SuperScript IV Reverse Transcriptase enzyme (Thermofisher Scientific, MA, USA), 1 µL of dithiothreitol (DTT, Thermofisher Scientific, MA, USA), 1 µL of RNase inhibitor (Promega) and 4 µL of 5x First Strand Buffer (Thermofisher Scientific, MA, USA) to bring the sample volume to 20 µL. The samples were then added to the T100 thermal cycler and incubated at 23°C for 10 min, followed by 53°C for 10 min and 80°C for 10 min. Samples were then maintained on ice or at 4°C or frozen at either −20 °C for short term use or −80 °C for long term storage. 1 μL of each cDNA sample was added to the well of a clear 96 well plate alongside 10 μL of Brilliant III Ultra-Fast SYBR Green qPCR Master Mix (Agilent Technologies, CA, USA), 0.4 µL of the primer for the target gene of interest (containing equal volumes of forward and reverse primers for each gene at a concentration of 0.2 μM) and 8.6 μL of Ambion™ nuclease free water (Thermofisher, MA, USA), bringing the total volume per well to 20 μL. The Brilliant III mixture contains Taq DNA polymerase, and a SYBR Green fluorescent dye yielding a fluorescent signal in proportion to the amount of DNA present. The plate was protected from light throughout and sealed with a clear adhesive film (Thermofisher, MA, USA). The plate was briefly centrifuged to eliminate bubbles and encourage mixing and inserted into a CFX Connect RT-PCR machine (Bio-Rad, CA, USA).

Each sample was quantified for the expression of Type II Collagen (*COL2A1*), *SOX9,* Aggrecan (*ACAN*) and Type I Collagen (*COL1A1*) relative to the geometric mean of two reference genes (*RPL13A* and *TBP*). Melt curves were inspected for each primer used. Each material was harvested for RNA extraction and PCR analysis at 4 h (Day 0), 24 h (Day 1), 7 days and 14 days of culture. All relative gene expression values were expressed as fold-changes using the ΔΔCt method compared to a control value: in this case the static condition at Day 0. Primers were designed using Primer-BLAST software (NIH, USA) with a view to yield primers specific for human genes, spanning exon-exon boundaries. The primers selected were purchased from Sigma Aldrich (now Merck) or Eurofins genomics (Ebersberg, Germany) and initially screened against the NCBI BLAST database to ensure homology to other genes was avoided. The primer sequences used in this research are available in Table 1 and were unmodified.

**TABLE 1:** Primer sequences for genes anaylsed using qPCR.

Gene		Primer sequence	Accession number	Product size (base pairs)
*TBP*	Forward	5′-GCC​AAG​AGT​GAA​GAA​CAG	NM_001172085.2	90
	Reverse	5′-GAA​GTC​CAA​GAA​CTT​AGC​TG		
*RPL13A*	Forward	5′-GTC​TGA​AGC​CTA​CAA​GAA​AG	NM_012423.4	189
	Reverse	5′-TGT​CAA​TTT​TCT​TCT​CCA​CG		
*ACAN1*	Forward	5′-CAC​CCC​ATG​CAA​TTT​GAG	NM_001135.4	82
	Reverse	5′-AGA​TCA​TCA​CCA​CAC​AGT​C		
*COL2A1*	Forward	5′-GAA​GAG​TGG​AGA​CTA​CTG​G	NM_001844.5	165
	Reverse	5′-CAG​ATG​TGT​TTC​TTC​TCC​TT		
*SOX9*	Forward	5′-CTC​TGG​AGA​CTT​CTG​AAC​G	NM_000346.4	172
	Reverse	5′-AGATGTGCGTCTGCTC		
*COL1A1*	Forward	5′-GGA​CAC​AGA​GGT​TTC​AGT​GGT	NM_000088.4	185
	Reverse	5′-GCA​CCA​TCA​TTT​CCA​CGA​GC		

### 2.5 Quantification of extracellular matrix deposition

The Dimethylmethylene Blue (DMMB) assay was used to quantify total glycosaminoglycan (GAG) amount in each material at 7 days and 21 days of culture. A stock solution of dimethylmethylene blue (DMMB) was made by dissolving 32 mg of 1,9-DMMB in 20 mL of pure ethanol overnight on an orbital shaker at room temperature. This stock solution was added to a mixture of 1.5 L distilled water, 59 mL 1 M NaOH and 7 mL 98% formic acid and left to mix for 2 h. The pH of the dye was adjusted and verified to be pH 1.5 prior to use. The cell-laden biomaterial hemispheres of each bioink were lysed in Radioimmunoprecipitation Assay (RIPA) buffer (Thermo Fisher Scientific, MA, USA) to yield protein isolates for GAG quantification along with material only control samples. Isolates were diluted 1 in 50 with distilled water and 40 μL added to the wells of a 96 well plate in triplicate with 200 μL DMMB reagent per well. The plates were read immediately at 525 nm compared to a series of chondroitin standards ranging from 0 to 50 μg/mL (0.03–0.75 μg). The total content of glycosaminoglycan in each sample was acquired using the standard curve of chondroitin samples, corrected for sample volume and dilution.

### 2.6 Histological analysis

The constructs were immersed in 4% paraformaldehyde (PFA) for 30 min in a 1:10 volume:PFA ratio (i.e. 1 mL PFA for a 100 μL biomaterial construct) on ice, washed with PBS and immersed in 1 mL of 30% (w/v) sucrose solution (Sigma Aldrich, MO, USA) until the constructs were fully permeated. The specimens were then immersed in Optimum Cutting Temperature (OCT) compound for 30 min and snap frozen in liquid nitrogen. Cryopreserved samples were mounted onto the plates of a Cryostat (Leica Biosystems, Wetzlar, Germany) using OCT and sectioned into 8–10 μm thick slices on to poly-L-lysine coated slides.

The mounted specimens were stained with 1% (w/v) Alcian Blue (Sigma Aldrich, MO, USA) and incubated at room temperature for 15 min, followed by a 1-minute incubation with Nuclear Fast Red Solution (Abcam, Cambridge, UK). Alternatively, slides were stained with picrosirius red stain (0.1% w/v, Sigma Aldrich, MO, USA) and left to incubate for 1 h at room temperature and washed with acetic acid solution. All stained specimens were covered in mounting medium (Histochoice, VWR, Pennsylvania, USA). Images were obtained from triplicates at 4x, 10x and 20x magnification using an Olympus CKX53 inverted microscope and Olympus CellSens software (Olympus, Japan).

### 2.7 Cell proliferation and viability

The alamarBlue assay (ThermoFisher Scientific, MA, USA) was used to provide an indication of cell number and metabolic activity. A 10% (v/v) alamarBlue solution was made by adding the solution to chondromedia, which was added to the cell-laden biomaterials for 4 h in standard culture conditions. Serial readings of 100 μL were taken at days 1, 7 and 14. The samples were added to separate wells of a 96 well plate with unreacted alamarBlue media added in triplicate to serve as control values. The degree of fluorescence was quantified using a plate reader (POLARstar Omega spectrophotometer, BMG LABTECH, Ortenberg, Germany) in which fluorescence intensity readings were taken using 570 nm (excitation) and 600 nm (emission) wavelengths, corrected for media only controls.

A live dead mammalian cell viability assay kit (Thermofisher Scientific, MA, USA) was used to visualise live and dead cells within the biomaterials. Media was discarded from the cells and pellets and washed in PBS. A 1:1000 Calcein-AM dye and 1:500 Ethidium homodimer-1 solution was added for 45 min, protected from light, and washed with PBS. The constructs were visualised using inverted fluorescent microscopy for live and dead cells at 4x and 10x magnification and 3 separate images per sample were acquired and counted using ImageJ software (National Institute of Health, USA).

### 2.8 *In silico* simulation of orbital cell shaking system

**FIGURE 1:**
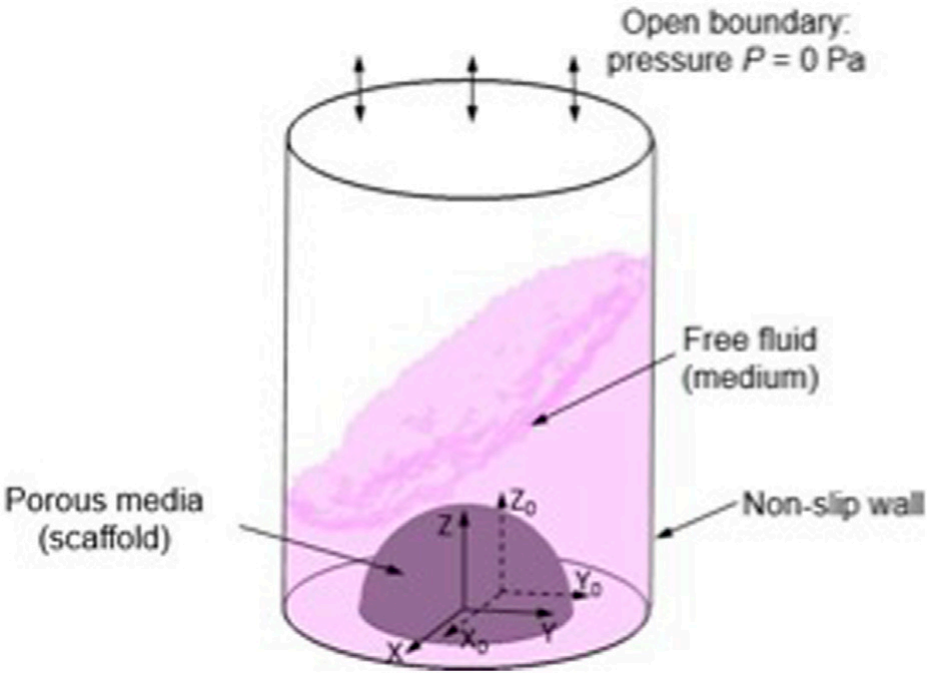
Illustration of CFD model setting and boundary conditions, (X, Y, Z) is the local coordinate and (X_0_, Y_0_, Z_0_) is the original coordinate.

To simulate the micro-mechanical environment generated within a crosslinked, cell-laden hydrogel (in this case nanocellulose-alginate) a multiphasic computational fluid dynamics (CFD) approach was used in this study as previously developed (Zhao et al., 2018; Melke et al., 2020). In the CFD model, a hemisphere with a volume of 100 µL was created to represent the cell-laden hydrogel droplet (Figure 1). The medium volume in each well was 0.75 mL, mirroring the experimental condition. In the CFD model, the cell-laden construct is modelled as homogenous porous media, of which the permeability was determined using the following Eq. 1 (Guyot et al., 2015).

$$\kappa =0.491∙\delta^{2}{\left(\frac{1-\psi_{c}}{1-\psi }-1\right)}^{1.155}$$ (1)

where, *Ψ*_*c*_ is the percolation threshold, *Ψ*_*c*_ = 0 (Guyot et al., 2015); *Ψ* is the porosity of the cell-laden hydrogel, *Ψ* = 42% (Al-Sabah et al., 2019); average pore size *δ* = 0.8 µm (Al-Sabah et al., 2019); therefore, *κ* = 2.1645 × 10^−13^ m^2^.

The medium in this model is assumed to be a Newtonian fluid with the density and dynamic viscosity of 1000 kg/m^3^ and 1 mPa/s, respectively (Maisonneuve et al., 2013). The top surface of the 48 plate well was defined as open boundary with the relative pressure of 0 Pa, and the side and bottom walls are defined as non-slip walls (Figure 6). The 48 well plate, in the coordinates of (X, Y, Z) was loaded with an orbital shaking speed of 500 rpm with reference to the coordinate of (X_0_, Y_0_, Z_0_). The Volume of Fluid technique was used in this CFD model for tracking the medium-air interface during shaking. For each element at the interface, the continuity and momentum equations were solved on the modified definition of the fluid properties (*P*) as per Eq. 2 (Salek et al., 2012):

$$\left\{\begin{array}{c} P=\alpha_{M}∙P_{M}+\alpha_{A}∙P_{A}\\ \alpha_{M}+\alpha_{A}=1 \end{array}\right.$$ (2)

where, *P*_*M*_ and *P*_*A*_ are the properties of medium and air that are density and dynamic viscosity; while *α*_*M*_ and *α*_*A*_ are the volume fraction of medium and air, respectively.

Both mixed fluid-air domain and porous media domain were meshed by a tetrahedron method with a patch-conforming algorithm. Transient analysis was used in the simulation with a time step of 0.006 s for 5 periods. Finally, the CFD model was solved by finite volume method using ANSYS CFX (ANSYS Inc, PA, USA) under the convergence criteria of root-mean-square residual of the mass and momentum <10^−4^.

### 2.9 Statistical analysis

Data sets were assessed for normality (Gaussian distribution) visually and where needed using an Anderson-Darling test. Statistical analysis were thereafter selected accordingly. All data presented is the mean value of three technical replicates from three separate patients. The mean data for biological replicates data are pooled and presented graphically with error bars depicting standard deviation unless otherwise stated.

The relative gene expression data sets were expressed relative to a control population (static conditions at Day 0) using the ΔΔCt method, these values were thereafter compared across conditions and time points using a 2 way ANOVA with Tukey’s *post hoc* multiple comparison test. A two-way ANOVA was used with a Tukey’s *post hoc* test for multiple comparisons was also used to assess for significance between live dead assays, the DMMB assays and alamarBlue assays between conditions and timepoints.

## 3 Results

### 3.1 CFD modelling and simulation

The cell-laden biomaterial hemispheres were subjected to dynamic orbital shaking at 500 rpm over the course of 14 days to determine the effects on chondrogenesis and to model the degree of mechanical stress this model exerted on the material and cells.

**FIGURE 2:**
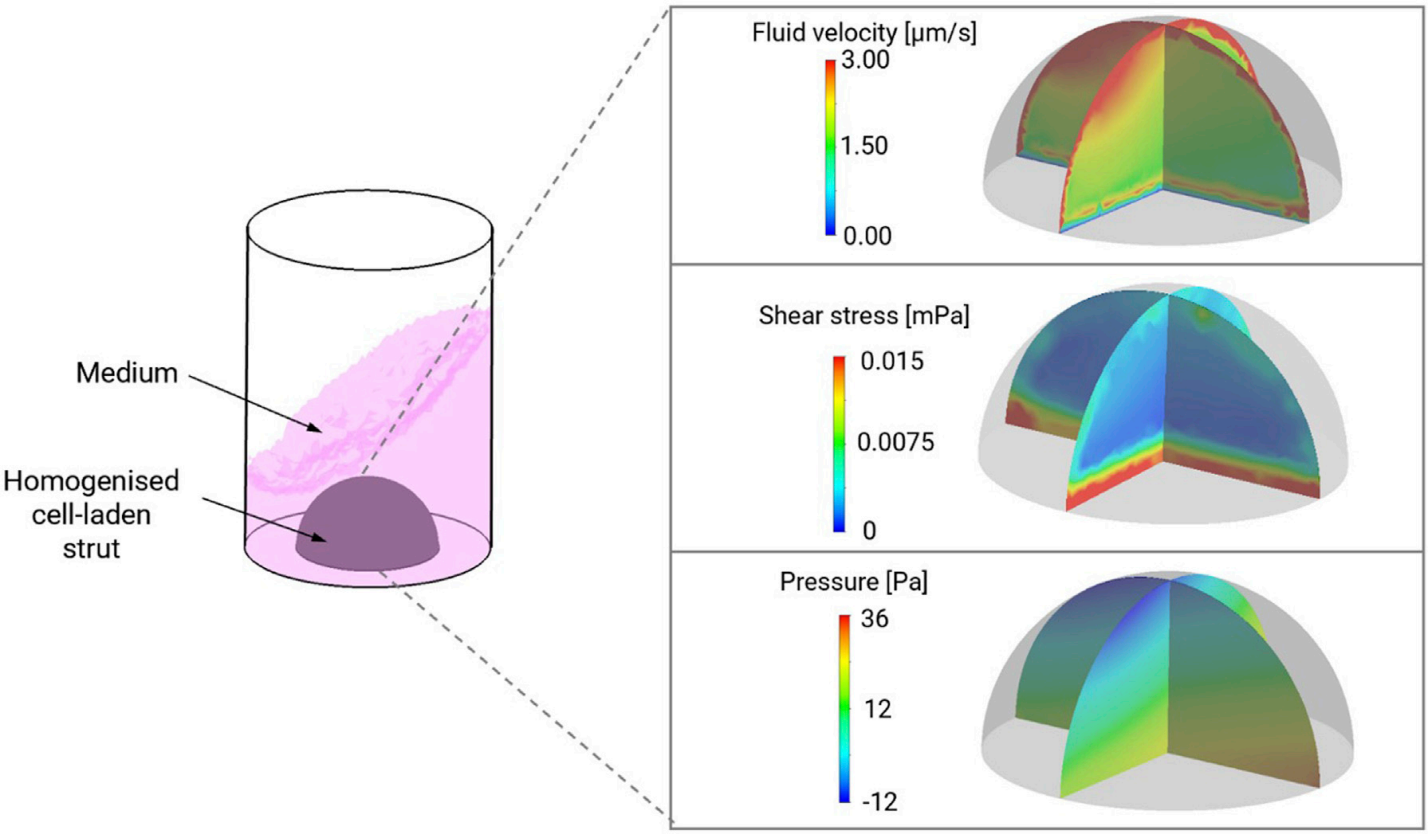
Computation modelling of shear stress, pressure and fluid velocity in NCB-alginate biomaterials with chondrocytes in dynamic culture conditions in a 48 well plate. Maximum fluid velocity was predicted in the upper poles of the material, with the greatest amount of shear stress at the base.

*In silico* simulation identified that the maximum fluid velocity experienced by the material-cell combination was under 3 μm/s with a maximum pressure range of 48 Pa and shear stress less than 0.02 mPa, with the most intense shear stress predicted at the base of the hemisphere (Figure 2).

### 3.2 Chondrogenic gene expression under dynamic culture conditions

**FIGURE 3:**
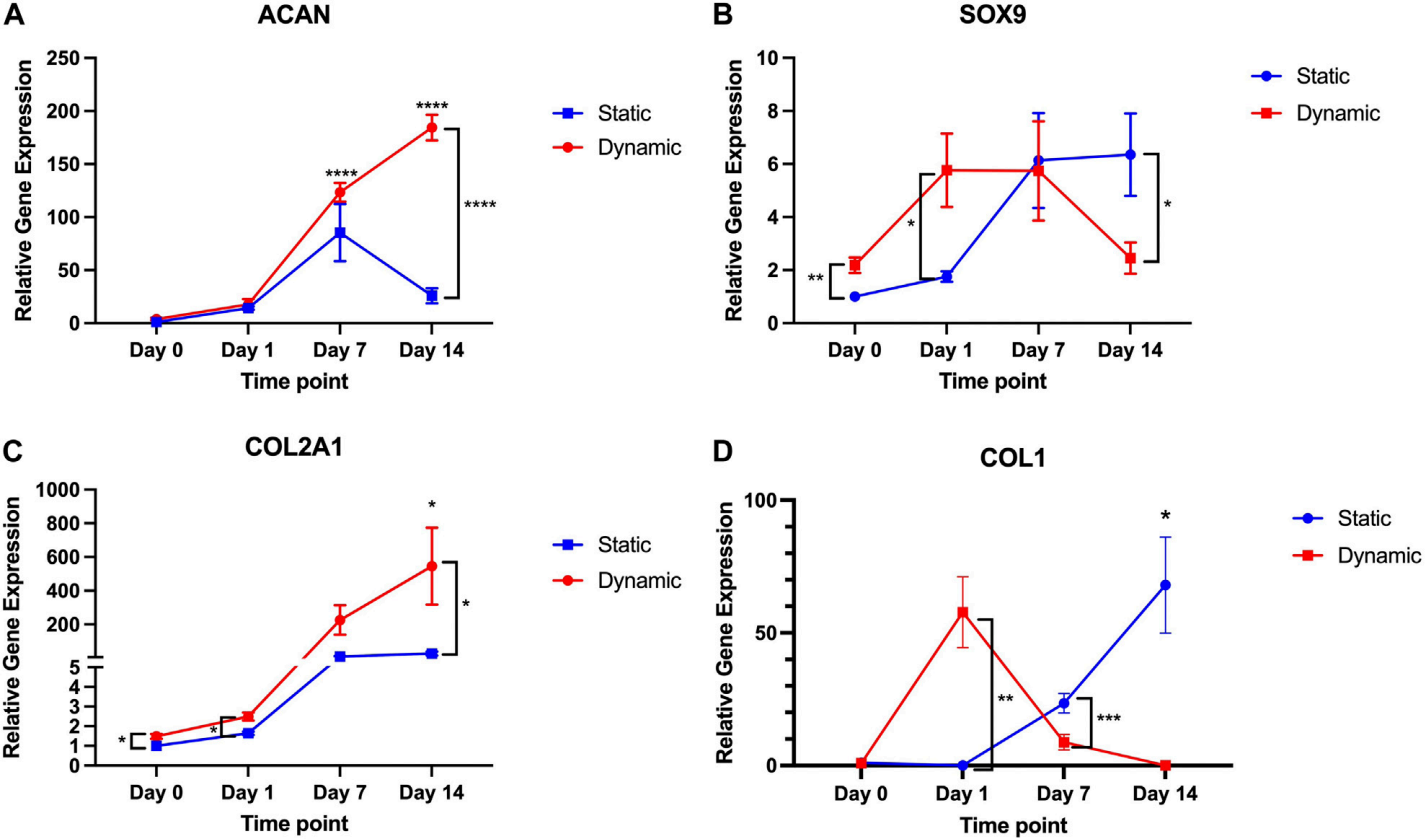
*ACAN, SOX9, COL2A1* and *COL1A1* gene expression over 14-day time period in dynamic and static culture conditions (*n* = 6–3 patients, technical duplicates). Values of the mean gene expression of the chondrogenic gene markers **(A)** *ACAN*, **(B)** *SOX9*, **(C)** *COL2A1* and **(D)** *COL1A1* are expressed relative to static conditions at Day 0 (as control) with standard error. * = *p* < 0.05; ** = *p* < 0.01; *** = *p* < 0.001; **** = *p* < 0.0001.

Despite the low levels of pressure and shear stress exerted in this model, there were enhancements in chondrogenesis observed with the dynamic culture environment. This trend was visualised through observing the gene expression profile over a 14-day timepoint series in dynamic and static culture conditions (Figure 3). Specifically, at Day 0 and Day 1, 7 and 14 days, the relative gene expression of *ACAN, SOX9, COL1A1* and *COL2A1* was calculated as a fold-change in gene expression relative to control values (static, Day 0) and compared across time points and conditions. Aggrecan expression rose significantly as early as Day 7 in static (85.4-fold increase) conditions compared to Day 0 but began to decrease by 14 days in the static culture conditions (25.9-fold increase), at no time point was *ACAN* expression significantly different to the initial Day 0 time point. Conversely, in the dynamic conditions *ACAN* expression remained elevated at both 7 days (123.5-fold increase, *p* < 0.0001) and 14 days (184.5-fold increase, *p* < 0.0001). At 14 days, the *ACAN* expression was significantly greater than Day 14 expression in the static conditions (*p* < 0.0001). In the dynamic conditions, a disparity in *SOX9* expression was seen as early as Day 0 (2.2-fold increase) compared to static conditions (*p* = 0.0097), followed by a transient rise in *SOX9* expression observed at Day 1 (5.6-fold increase, *p* = 0.03 versus static Day 1) and 7 days (5.7-fold increase) but fell thereafter at 14 days (2.4-fold increase, *p* = 0.048 compared to static Day 14) indicating an early, transient signal towards chondrogenic differentiation in the dynamic group. Conversely, a more gradual increase in *SOX9* expression was observed over 14 days in the static conditions, peaking at a 6.7-fold increase at 14 days. Conversely, with regard to *COL2A1*, there was a marked increase in expression at Day 1 (2.5-fold, *p* = 0.03 versus static at Day 1) and 14 days (546.6-fold increase, *p* = 0.038 compared to static Day 14) in the dynamic culture conditions relative to static conditions. Although increases in *COL2A1* gene expression were also observed in the static conditions these were not significant increases across the timepoints studied. To determine whether there was fibrocartilage production, *COL1A1* expression was also examined in the dynamic and static conditions. There was a transient rise in *COL1A1* expression in the dynamic culture conditions relative to static conditions at 4 h (57.8-fold increase) but this was not statistically significant. Thereafter, there was a fall in *COL1A1* expression in dynamic conditions to the extent that at day 14 no detectable *COL1A1* gene expression was identified in any biological or technical repeat. In static conditions, a gradual increase in *COL1A1* expression was observed over the 14-day time period, peaking at a 68-fold increase at Day 14 (*p* = 0.02). *COL1A1* expression was also significantly higher than dynamic conditions at Day 7 (*p* < 0.001).

This data indicates that dynamic culture conditions evoke a transient statistically significant increase in *SOX9* and *COL1A1*, followed by sustained increases in *ACAN* and *COL2A1* expression and along term attenuation of *COL1A1* expression.

### 3.3 Extracellular matrix production and deposition under dynamic culture conditions

**FIGURE 4:**
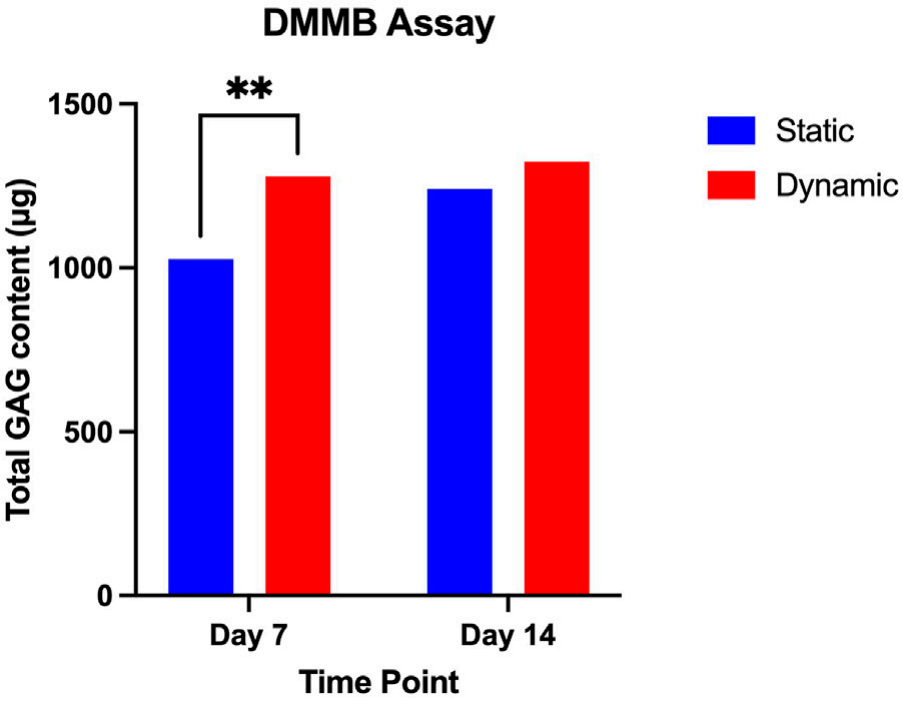
DMMB Assay comparing mean GAG content of static and dynamic culture conditions at day 7 and day 14 of culture. Mean values at each time point are presented with standard deviation and pairwise comparisons from a 2-way ANOVA (data from three biological repeats, *n* = 9). * = *p* < 0.05.

With regard to the production of extracellular matrix (Figure 4), there were no significant differences in the quantity of glycosaminoglycan produced between the two culture conditions at 14 days (*p* = 0.8), but significant differences were noted between the static conditions at Day 7 (1027 µg) and the dynamic conditions at day 7 (1,279 μg, *p* = 0.009).

Whilst differences between the dynamic and static conditions are noted at the level of gene expression, this has manifested in differences in glycosaminoglycan production primarily at Day 7 in the dynamic conditions. This supports the hypothesis that the enhanced perfusion through the biomaterial in the dynamic culture conditions may have contributed to accelerated extracellular matrix deposition.

**FIGURE 5:**
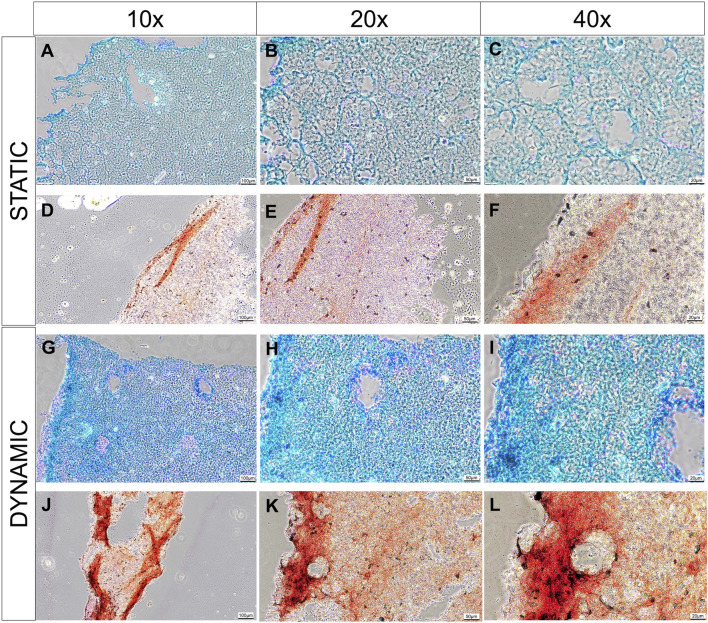
Histological analysis of cell-laden biomaterials after 14 days of culture, stained with Alcian Blue stain and counter stained with nucelar fast red stain in static **(A–C)** and dynamic **(G–I)** conditions; or stained with picorsirius red in static **(D–F)** or dynamic culture conditions **(J–L)**. Images are taken at 10x **(A, D, G, J)**, 20x **(B, E, H, K)** and 40x **(C, F, I, L)** magnification. Scale bars are present in the bottom right of each image depicting 100 µm **(A, D, G, J)**, 50 µm **(B, E, H, K)** and 20 µm **(C, F, I, L)**. Representative images from 3 technical repeats are presented.

Histologically, there is evidence that greater pericellular extracellular matrix deposition was occurring in the dynamic culture conditions compared to the static conditions (Figure 5). A greater intensity of Alcian blue stain is visible in the dynamic culture conditions at the end of the experimental period (14 days) which appears to be more pronounced at the periphery of the biomaterial (Figures 4G–I), correlating to the areas in which greatest fluid velocity would be anticipated using the *in silico* modelling (Figure 1A). Conversely, weaker and more uniform Alcian blue staining was visualised in the static culture conditions, with more pronounced pores devoid of extracellular matrix. A similar phenomenon was observed with collagen deposition stained with Picrosirius red (Figures 4J–L): a greater degree of stain was noted at the periphery of the hemisphere on both the flat and convex surface of the construct (Figure 4J), and the staining was more pronounced in the dynamic culture conditions than the static conditions.

### 3.4 Cell viability and proliferation

**FIGURE 6:**
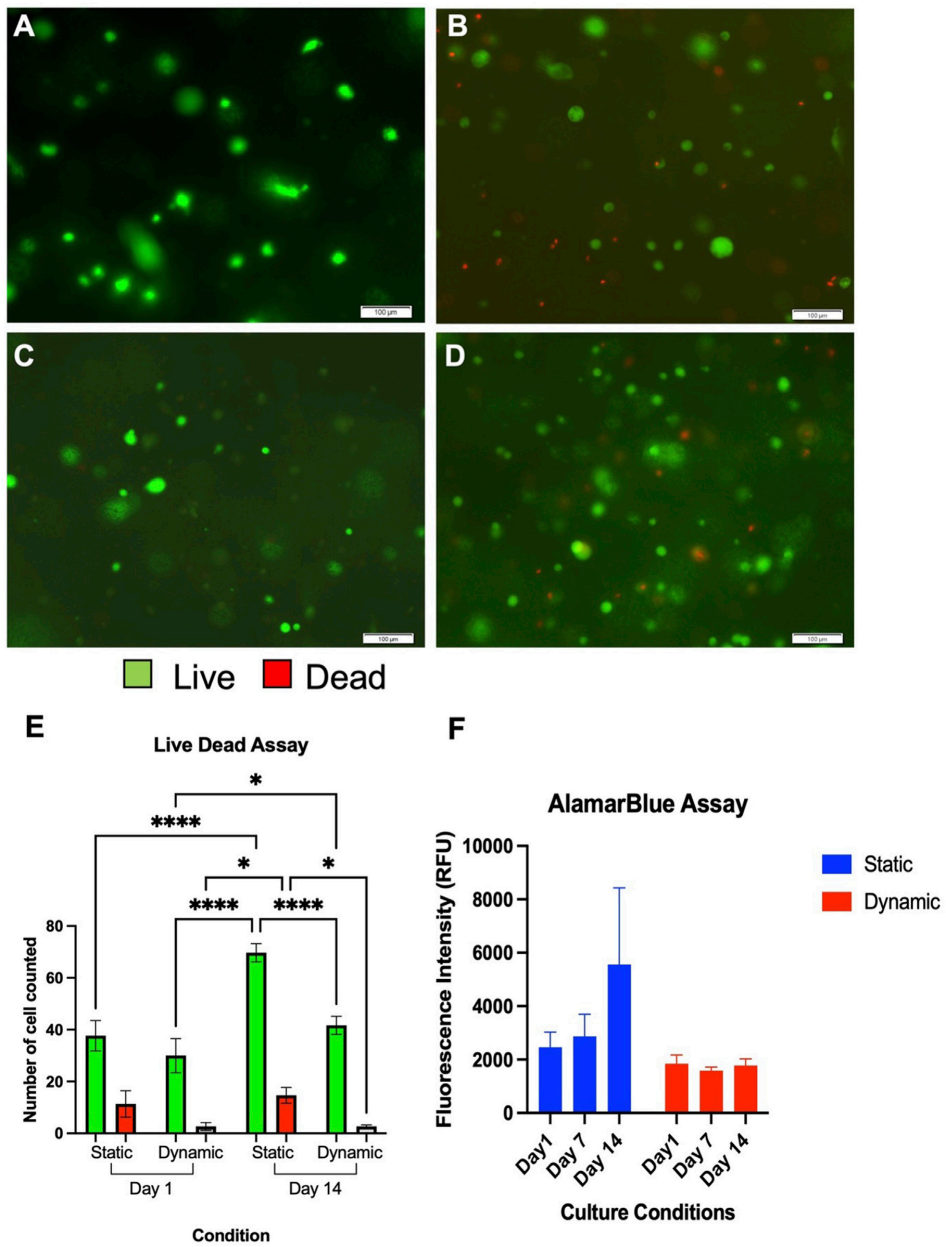
Live Dead Assay of chondrocytes cultured in NCA in static **(A+C)** and dynamic **(B+D)** settings at 24 h **(A+B)** and 14 days **(C+D)**. Live cells are coloured green and dead cells are coloured red. Scale bars denote 100 µm. A significantly higher number of chondrocytes were counted in the static conditions albeit adequate viability (>80%) is observed in both conditions at 14 days **(E)**. Data depicts three technical repeats. **(F)** AlamarBlue Assay demonstrating comparable fluorescence in each condition and time point from three technical repeats. RFU, Relative Fluorescence Units.

To determine the effects of orbital shaking on cell proliferation and viability, live-dead and alamarBlue assays were undertaken (Figure 6).

Using the live-dead assay, there were no significant differences in cell numbers seen between the two samples at the initial time point (*p* = 0.15, Figures 6A–E). Visible increases in cell number were observed with both conditions over the 14 days culture period however, and these were significant increases in both the static conditions between Day 1 and Day 14 (*p* < 0.0001) and in the dynamic conditions between Day 1 and Day 14 (*p* = 0.017). In the areas analysed, a significantly greater number of cells were counted in the static conditions at Day 14 compared to dynamic conditions (*p* = 0.014). However, despite higher numbers of cells being present in the static conditions at 14 days, the percentage viability was a mean of 77.6% ± 5.6% at Day 1 in static settings and 91.5% ± 5.9% in dynamic conditions (*p* = 0.011). At Day 14, the mean viability remained higher in the dynamic conditions at 94.0% ± 1.4% compared to 82.6% ± 3.7% in static conditions (*p* = 0.030).

The primary findings of the alamarBlue assay (Figure 6F), were that greater cell numbers were implied from the fluorescence of the static conditions at 14 days, with the greatest mean fluorescence intensity at this time point (5550 RFU). However, the differences between dynamic and static culture conditions were not significant at any time point, and moreover did not appear to significantly change over the 14 days experimental period. This data, combined with the differences in gene expression and ECM production, may indicate that the dynamic conditions promote chondrogenic differentiation rather than proliferation. It can be concluded, therefore, that whilst orbital shaking culture conditions may enhance chondrocyte viability and chondrogenicity, there is no perceived benefit with regard to enhanced cellular proliferation.

## 4 Discussion

In this study, we explored the role of an orbital shaking system as a simple means of enhancing the culture of tissue engineered cartilage. This set up sought to recreate and simplify the advantageous effects of bioreactor culture using basic laboratory equipment, and quantify the impact on chondrogenesis. This study demonstrates novelty in the use of dynamic culture to enhance human nasoseptal cartilage biofabrication and in the coupling of tissue engineering and *in silico* modelling to characterise whether any contributing mechanical stimulation might underpin the observed effects.

There were significant enhancements in chondrogenic gene expression achieved through the orbital shaking system, although this did not translate to biochemically greater levels of GAG being detected, extracellular matrix deposition was noted particularly in the periphery of the cell-biomaterial construct and microscopically in a pericellular location on histological analysis. This mirrors findings seen in bioreactors used to enhance articular cartilage tissue engineering, whereby dynamic culture conditions, specifically in the form of shear and mechanical compression stresses, were associated with greater chondrogenic gene expression (Guo et al., 2016). Similarly, previously investigated dynamic culture models include the use of a linear “see-saw” plate shaker, which has been shown to promote chondrogenic differentiation of induced pluripotent stem cells with increased toluidine blue staining, cell clustering and augmented expression of chondrogenic genes *COL2A1*, *ACAN* and *SOX9* at frequencies of 0.3–0.5 Hz (Limraksasin et al., 2020). In the latter study, this mechanism was believed to be attributable to an upregulation of TGF-B and Wnt signalling genes, directing the stem cells down a chondrogenic differentiation pathway (Limraksasin et al., 2020). There is evidence however, that although the see-saw approach may be beneficial for directing iPSCs down a chondrogenic lineage, bovine articular chondrocytes seeded in an agarose gel and cultured in an orbital shaking system had superior Young’s modulus, GAG content and collagen content compared to those cultured in uniaxial rocking conditions (Cigan et al., 2014). Our study complements the literature in the field in this regard, expanding our knowledge further through an assessment of the temporality of chondrogenic gene expression in short term culture. We demonstrate an early spike in *SOX9* gene expression achieved in the first 24 h of dynamic culture is likely to translate to superior sustained *COL2A1* and *ACAN* gene expression throughout the remainder of the culture period, with a reduction in *COL1A1* expression from 7 days in dynamic culture.

The media used in this study was intentionally basic, containing only media constituents required for cell metabolism and ECM production, but not supplemented with growth factors to direct differentiation or proliferation. In doing so, the inherent chondrogenic potential of the culture conditions can be ascertained. There are limitations to the use of simplistic media constituents however, such as dedifferentiation, fibrocartilage synthesis and attenuated cell proliferation. In this studt, there was a steady increase in fibrocartilaginous gene expression observed in static conditions and the AlamarBlue study failed to adequately demonstrate absolute cell numbers by assessing cell metabolism at the end of the study. However, in future applications, the augmentation of media with growth factors would certainly enhance the proliferation further as demonstrated in previous studies, coupled to more reliable means of assessing total cell number (Iwasaki et al., 1993; Tonon and D’Andrea, 2000; Ogueta et al., 2002; Heng et al., 2004).

There have been a number of significant developments in the field of 3D bioprinting using nasoseptal chondrocytes and indeed of using dynamic culture conditions for optimizing articular chondrogenesis (Martín et al., 2016; Schwarz et al., 2020; Lan et al., 2021). Whilst the advantages of dynamic culture have been described previously, these have been in the context of articular cartilage tissue engineering and nonetheless, the mechanisms by which enhanced chondrogenesis are achieved remain incompletely understood (Cigan et al., 2014; Shahin and Doran, 2015; Limraksasin et al., 2020). As a load bearing cartilage, the effects of compressive loading, hydrostatic pressure and shear on chondrogenic gene expression in articular cartilage have been well explored but this mechanotransducive threshold has not been as extensively explored in the facial cartilages (Takano-Yamamoto et al., 1991; Princz et al., 2017). In articular cartilage tissue, intermittent hydrostatic pressures of as high as 10 MPa have been required to evoke increases in cartilage gene expression (*SOX9, COL2A1, ACAN*) (Fahy et al., 2018) or shear stresses of 0.1 Pa (Gemmiti and Guldberg, 2009). Through the use of *in silico* modelling, we were able to demonstrate that the degree of shear stress within the scaffold was low (a maximum of 0.02 mPa) and approximately 10000-fold lower than the shear stress required to evoke a chondrogenic gene change in articular cartilage (Gemmiti and Guldberg, 2009). It is additionally unlikely that pressure gradients of 48 Pa evoked by the orbital shaking system would be sufficient to evoke a mechanotransductive signal, as this is in the region of 200,000-fold lower than that of articular cartilage (Fu et al., 2021). As such, the pro-chondrogenic effect appeared to be independent of shear stress and pressure. A more likely explanation of the enhanced chondrogenesis is attributable to the porosity of the scaffold and the augmented perfusion of media throughout the scaffold as depicted by the enhanced fluid velocity through the scaffold (Figure 1). This phenomenon has also been observed in comparisons of chondrogenesis in static and dynamic conditions (a perfused bioreactor), where flow rates of 1 μm/s demonstrated significant elevations in DNA content, GAG content and hydroxyproline content compared to static conditions (Pazzano et al., 2000), and a study of nasoseptal chondrocytes cultured in decellularized porcine cartilage where enhanced cellular migration and aggrecan synthesis in an automated perfusion bioreactor environment were observed, mirroring the gene expression enhancement seen in this study (Princz et al., 2017). Similarly, enhanced flow through the introduction of perfusion channels was demonstrated to increase chondrogenesis in dynamic culture in one study (Cigan et al., 2014). It is possible therefore that flow speeds of 3 μm/s were sufficient to evoke an elevation in chondrogenesis in this study, particularly as we have previously demonstrated the nanocellulose-alginate biomaterials used in this study demonstrate high porosity (Kyle et al., 2018; Al-Sabah et al., 2019). In addition to increasing the delivery of nutrients through enhanced media perfusion, it has been previously reported that fluid flow has a key role in chondrocyte biosynthesis (Zhao et al., 2020), with postulated mechanisms being attributed to mechanosensitivity sensed by PIEZO calcium channel receptors (Lee et al., 2014), the primary cilium (Yuan and Yang, 2015) and integrins (Zhang et al., 2008). The impact of orbital shaking on chondrogenesis is therefore potentially multifactorial and may be attributable to flow-mediated mechanotransductive signals that warrant further investigation. In particular, the effects of mechanical stimuli in nasoseptal cartilage tissue engineering remain considerably less well explored compared to their articular counterparts, but will have implications for the mechanical stresses of 3D bioprinting in addition to optimizing tissue maturation (Schwarz et al., 2020). Owing to its roles as a non-load bearing cartilage and its embryological origin from neural crest cell derivatives, it is unlikely that findings in articular cartilage can be translated directly to nasoseptal cartilages (Pelttari et al., 2017). After cementing a foundation of knowledge in this area, there is potential to exploit these mechanotransductive pathways for the purposes of enhancing tissue engineering and bioreactor design to facilitate superior biomimicry and augment the translational potential of tissue engineered cartilage.

## 5 Conclusion

In this study we have highlighted that an orbital shaking system can be implemented to enhance the chondrogenicity of tissue engineered cell-biomaterial constructs. The novelty of this study lies in the use of human nasoseptal chondrocytes and a correlation of the biological effects of orbital shaking with an *in silico* approach, to estimate the effects of mechanical stimuli on nasoseptal chondrogenesis. Whilst evidence of augmented chondrogenic potential was observed both histologically and at the level of chondrogenic gene expression, in addition to superior cell viability, this is unlikely to be a result of biologically significant mechanotransduction pathways, and represents enhanced perfusion of media through the porous biomaterial scaffold. Further investigation into the sensitivity of nasoseptal cartilage to mechanical stimuli is warranted to deepen our understanding of mechanotransducive thresholds and exploited for future nasoseptal cartilage tissue engineering efforts.

## Data Availability

The raw data supporting the conclusion of this article will be made available by the authors, without undue reservation.
